# Cardiac Tamponade Due to Inferior Vena Cava Filter Removal: A Case Report and Review of Literature

**DOI:** 10.7759/cureus.6996

**Published:** 2020-02-14

**Authors:** Sohaib Roomi, Shujaul Haq, Waqas Ullah, Munnam S Jafar, Mahnoor Sherazi

**Affiliations:** 1 Internal Medicine, Abington Hospital - Jefferson Health, Abington, USA; 2 Internal Medicine, Jinnah Hospital Lahore (JHL)/Allama Iqbal Medical College (AIMC), Lahore, PAK; 3 Internal Medicine, Fauji Foundation Hospital, Rawalpindi, PAK

**Keywords:** inferior vena cava, cardiac tamponade

## Abstract

Cardiac tamponade is a condition characterized by the accumulation of pericardial fluid, compromising the hemodynamics of the circulation. It has several known causes, including traumatic injury to the pericardium, idiopathic, neoplastic or purulent pericarditis, and, rarely, iatrogenic etiology. Inferior vena cava (IVC) filter removal can lead to multiple complications including but not limited to IVC perforation, air embolism, pneumothorax or filter migration. Here, we present a case of a middle-aged woman presenting with cardiac tamponade after IVC filter removal. She was successfully managed with pericardiocentesis followed by pericardial window placement. As this case and literature review illustrates, cardiac tamponade is a rare but potentially devastating complication of IVC filter manipulation.

## Introduction

Cardiac tamponade is a condition characterized by the pericardial fluid accumulating to compromise circulation hemodynamics. Cardiac tamponade can be caused by a number of different mechanisms including, but not limited to, mechanical trauma to the pericardium during chest wall injury, acute pericarditis, and myocardial rupture, especially after myocardial infarction or iatrogenic etiology. Inferior vena cava (IVC) filter manipulation can lead to IVC perforation, fracture or migration of IVC filter, air embolism, pneumothorax, insertion site thrombosis or wound infection. IVC filter manipulation leading to cardiac tamponade is very rare. Here, we present a case of a middle-aged woman presenting with cardiac tamponade after IVC filter removal. 

## Case presentation

A 49-year-old woman with a past medical history of pulmonary embolism presented for IVC filter removal, which was initially placed approximately three months prior to this hospitalization. She was not a smoker and had no history of chronic lung disease or occupational hazards. At that time, the patient had right leg deep vein thrombosis and right sub-segmental pulmonary artery embolism. Hematological workup was inconclusive and it was presumed to be provoked by prolonged bed rest in the setting of bariatric surgery. The patient had a history of intracranial aneurysm in the posterior circulation so long-term anticoagulation was not thought to be a safe option. She got IVC filter placed via the right femoral approach.

**Figure 1:**
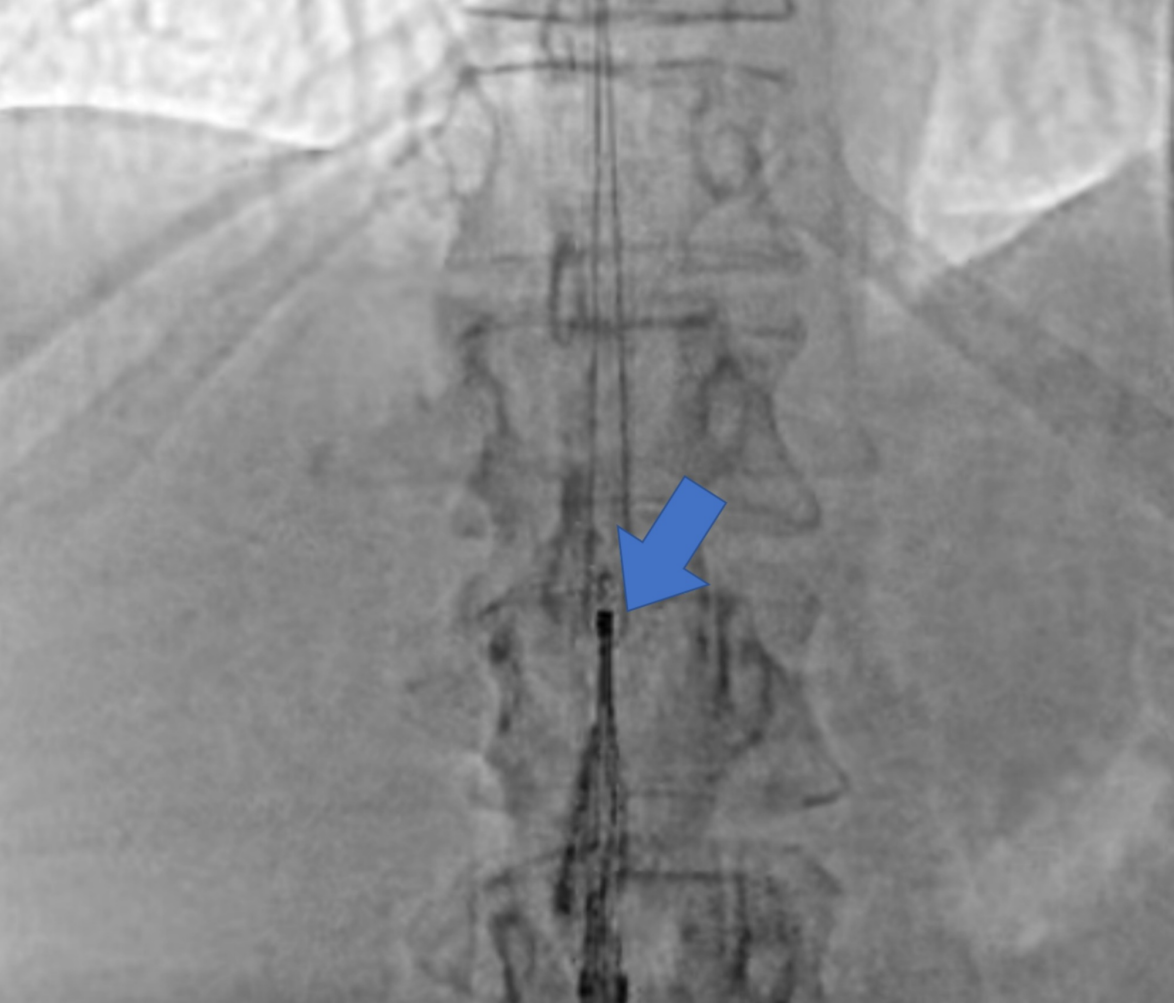
Fluoroscopy revealing inferior vena cava filter removal via snare

In the present hospitalization, interventional radiology (IR) guided IVC filter removal was done in the operating room (OR). Under X-ray fluoroscopy, a snare was inserted into the internal jugular vein, and the IVC filter was withdrawn from its hook (Figure 1). Per the OR notes, it was a difficult approach requiring manipulation of the snare. The patient was transferred after the procedure into an observation room.

**Figure 2:**
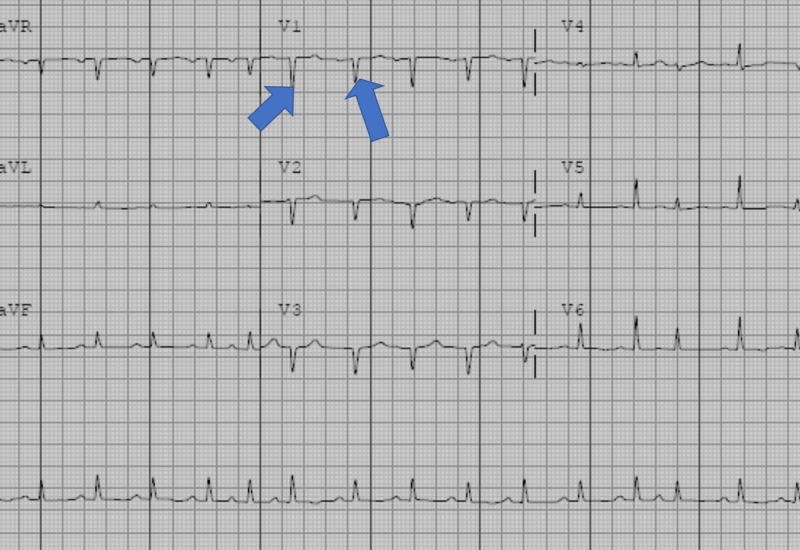
Electrocardiogram revealing sinus tachycardia, low voltage QRS complexes, and a prolonged QT interval Variation in  QRS amplitude with beats is called electrical alternans, a sign specific for cardiac tamponade. Arrows in the figure denote variation in QRS complex amplitude.

Within an hour after the procedure, she developed pleuritic chest pain, hypotension, presyncope, and shortness of breath. She denied any other systemic symptoms like nausea, vomiting, diarrhea, joint pain, and bowel or bladder symptoms. Her physical examination showed a blood pressure of 85/60 mmHg, a pulse of 122 beats per minute, a respiratory rate of 26 breaths per minute, and an oxygen saturation of 92%. She had a poor inspiratory effort. Her jugular venous pressure was elevated (9 cm of H2O). On cardiovascular examination, her heart sounds were markedly diminished, and the point of maximal impulse was nonpalpable. No murmurs, gallops, or rubs were appreciated. Her chest was clear on auscultation. The results of her abdominal and neurological examinations were also unremarkable. Her pertinent laboratory findings revealed PT/INR of 3.4, a potassium level of 3.1 mEq/L, and hemoglobin of 10.6 g/dL. The other laboratory tests were unremarkable. Her chest X-ray revealed low lung volumes with bibasilar subsegmental atelectasis. An electrocardiogram (EKG) revealed sinus tachycardia, electrical alternans, low voltage QRS complexes, and a prolonged QT interval (Figure 2).

**Figure 3:**
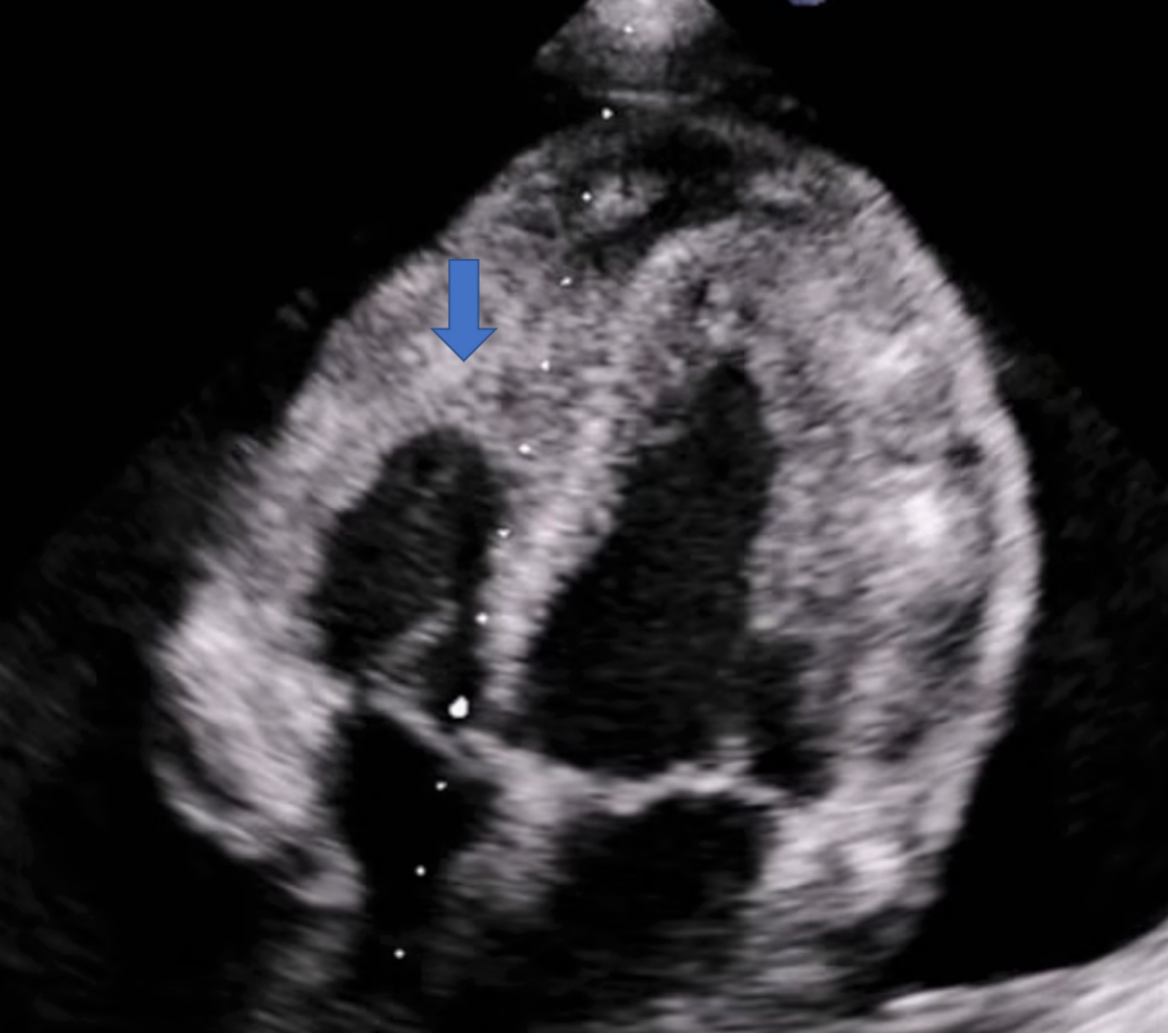
Echocardiogram showing the diastolic collapse of the right ventricle as indicated by the arrow in the figure

Bedside echocardiography (echo) at the symptom onset showed a large anterior, small to moderate lateral and posterior pericardial effusion. Left ventricular and right ventricular functions were grossly normal. Transthoracic echo revealed moderate to severe pericardial effusion, 2.1 cm in the largest dimension with right ventricular collapse during diastole consistent with the tamponade effect (Figure 3). It also showed a large echogenic mobile structure, consistent with pericardial thrombus.

 

She was started on intravenous fluid resuscitation and immediately transferred to the OR. With an emergent pericardiocentesis, 300 mL of blood and a large clot was removed, and a pericardial window was placed. Postoperative transthoracic echocardiography (TTE) revealed that pericardial blood volume was significantly improved with only a small amount left anteriorly. The pericardial thrombus was no longer evident. She remained in the hospital for two days after the procedure. The drainage catheter was removed on the second day when it drained less than 25 ccs of fluid over the day. She was subsequently discharged in a stable condition, and an outpatient follow-up was advised.

## Discussion

Cardiac tamponade occurs when pericardial fluid accumulates within pericardial space leading to reduced cardiac filling and, eventually, hypotension or shock. Cardiac tamponade has an estimated frequency of two per 10,000 and in 2% of chest gunshot wound victims [1]. Fluid accumulation within the pericardial space leads to a progressive decline in the cardiac filling. The resulting pericardial pressure forces the interventricular septum to bend towards the left side, which leads to a decrease in stroke volume [1]. We conducted a thorough search of the literature published to date with the search terms ‘vena cava filter’ and ‘cardiac tamponade’ on PubMed. We found a total of six published articles, which are summarized in Table 1 [2-7]. For this subset of patients, the average age is 48 years (range, 29 to 66 years) with a 5:1 ratio of male to female patients. The most common presentations were hypotension, tachycardia, and tachypnea in 66% of the population. About 16% of patients were asymptomatic, or there were not enough data mentioned. In 33% of the patients, chest pain and shortness of breath were the presenting concerns. Of these patients, 16% of patients had associated lower extremity weakness, and a similar percentage of patients had fatigue. Sixteen percent of the patients had an open skull fracture and multiple traumatic injuries incurred in a road traffic accident that caused them to seek immediate treatment leading to cardiac tamponade. History of deep venous thrombosis, pulmonary embolism, or both were present in 86% of the patient population. Deficiency of protein C, protein S or both were present in 33% of the patients. Other comorbidities included ulcerative colitis, hypertension, diabetes, and chronic kidney disease, each of them affecting 16% of the patient population. Cardiac tamponade in half of these patients was managed by pericardiocentesis followed by pericardial window placement and by cardiothoracic surgery in the other half. All patients recovered and were discharged in stable condition with 0% mortality. In five of these cases, cardiac tamponade occurred with IVC filter manipulation, and one case was associated with a filter in the superior vena cava.

**Table 1:** Characteristics of previously reported cases of inferior vena cava filter related cardiac tamponade f, female; m, male; IVC, inferior vena cava; SVC, superior vena cava; IR, interventional radiology; CKD, chronic kidney disease; Hx, History; DVT, deep venous thrombosis; PE, pulmonary embolism; SOB, shortness of breath

Author	Age/Sex/Filter Placement Location	Presentation	Vitals on Presentation	Comorbidities	Management	Outcome
Chou [3]	64/m/IVC	Fatigue	Not mentioned	protein C and protein S deficiency, Hx of DVT and PE	Percutaneous approach for stent removal failed, Laparotomy for filter removal, cardiothoracic surgery for tamponade	Resolved
Chandra [4]	53/m/IVC	Chest pain, SOB	Hypotensive, tachycardiac	Hypertension, diabetes, DVT, obesity	Sternotomy for removal of clot and fractured IVC filter	Recovered
Vergara [5]	35/m/IVC	SOB, lower extremity pain	Hypotensive, tachycardic tachypneic	Ulcerative colitis, PE, DVT	Percutaneous catheter removal, pericardiocentesis for tamponade	Resolved
Saeed [6]	66/f/IVC	Upper chest tightness, SOB nausea	Hypotensive, tachycardiac, tachypneic tachy, tachypneic	PE, DVT, cystocele	Sternotomy, IR guided filter removal	Resolved
Hussain [7]	f29/m/SVC	Open skull fracture, intracerebral hemorrhage, tibial shaft fracture	Hypotensive, tachycardiac, tachypneic	Multiple fractures, thrombosis of the brachial, axillary and subclavian vein	Pericardiocentesis, pericardial window	Resolved
Hsin [8]	45/m/IVC	Recurrent DVT, PE	Stable	Protein S deficiency, CKD, heart failure, cerebral infarction	Transthoracic echo guided pericardiocentesis	Resolved

Our case marks only the seventh reported case of this rare complication associated with IVC filter removal or trauma. We believe more such cases if published, will provide awareness for the management of this rare entity. Cardiac tamponade can be classified as acute or subacute depending upon the timeline and acuity of presentation or necessity of immediate intervention. Acute tamponade generally occurs within minutes, often resembles cardiogenic shock, and requires urgent intervention [8]. It is caused by trauma, aortic rupture, or can be iatrogenic from the therapeutic or diagnostic procedure. Symptoms typically include shortness of breath, chest discomfort, lightheadedness, cough, and fatigue. The rest of the symptoms are caused by an underlying cause [9]. Subacute cardiac tamponade generally has delayed presentation over days to weeks. It may be caused by idiopathic, infectious, autoimmune, or neoplastic pericarditis. A small subset of patients may have regional cardiac tamponade when localized hematoma or loculated pericardial effusion compresses only selected heart chambers. Our case was an example of an acute cardiac tamponade where the patient developed symptoms of shortness of breath, chest discomfort, and lightheadedness within one hour of IR-guided IVC filter manipulation via the jugular vein. We believe that this might have occurred due to the manipulation of the vasculature and possible migration of small IVC fragments. There was no evidence of right atrial myocardial rupture to suggest the iatrogenic rupture of the myocardium. The diagnosis of cardiac tamponade is made by a combination of physical signs and imaging. Dilated neck veins, muffled heart sounds, and low arterial pressure constitute Beck’s triad, which is present only in 10% to 40% of the patients having cardiac tamponade [10]. Imaging modalities most commonly employed include EKG, chest X-ray, and echo. EKG occasionally reveals sinus tachycardia, electrical alternans, and low-voltage QRS complex. Sinus tachycardia is more sensitive, but electrical alternans and low QRS voltage are specific for cardiac tamponade but rarely seen [9,11]. Findings on chest radiographs are relatively nonspecific and include enlarged cardiac silhouette with clear lung fields. At least 200 mL of fluid should accumulate in the pericardial space before it appears on the X-ray [9].

The echo is the diagnostic modality of choice. It shows the amount and location of pericardial fluid. In most patients with cardiac tamponade, echo reveals the diastolic collapse of the right atrium, right ventricle, or left heart collapse [12-13]. A prospective study on echo established sensitivity and specificity of 90% and 65%, respectively, for cardiac tamponade detection in patients with large pericardial effusion [14]. Computed tomography and cardiovascular magnetic resonance imaging are usually not needed when echo is available. In our case, the patient’s EKG showed sinus tachycardia and nonspecific T wave abnormalities. TEE revealed moderate to severe pericardial effusion, 2.1 cm in the largest dimension. It also showed a large echogenic mobile structure, consistent with pericardial thrombus, which is different from other cases included in the review. Management includes pericardiocentesis, cardiothoracic surgery, or conservative treatment depending upon the hemodynamic status of the patient [10]. In stable patients, careful monitoring of vital signs and serial echocardiograms is helpful. Fluid resuscitation can only be used to bridge the delay between pericardial fluid removal if the patient is unstable.

Pericardiocentesis involves catheter insertion under echo guidance at the best location, depending upon operator expertise, and left in place until fluid removal is less than 25 mL/day. Open surgical drainage, being more invasive, is performed under general anesthesia and preferred over pericardiocentesis when either pericardiectomy or pericardial biopsy is required or in cases where effusion is loculated. In a nonrandomized trial, percutaneous echo-guided pericardiocentesis resulted in lower complication and mortality rates compared to an open surgical approach [15]. After the procedure and initial stabilization, patients are observed with frequent vital monitoring, EKG, and echo to ensure adequate drainage of pericardial fluid. It is important to note that due to their hospitalization, these patients can still have a risk for venous thrombosis and pulmonary embolism. We believe that nonpharmacological management such as graduated compression stockings, intermittent pneumatic compression, and foot venous pump should be practiced in lieu of pharmacological management. In our patient, pericardiocentesis followed by the pericardial window was performed on both presentations, and a pericardial window was removed when less than 25 ccs of fluid was drained over 24 hours. Recurrence of pericardial effusion depends upon the etiology. The patient is usually discharged within 24 to 36 hours after stabilization and is advised to have a repeat echo as an outpatient to rule out any reaccumulation. In our patient, during the first admission, a pericardial catheter was kept in place for 30 hours and was removed when it drained less than 25 ccs/day. She got readmitted with pericardial effusion, and pericardial catheter drained over 300 cc fluid for 72 hours. Given that there are only a few published cases of cardiac tamponade resulting from IVC filter removal, there are no established guidelines for steps of management to date. More cases need to be reported so that data-driven guidelines can be established to manage this potentially devastating complication.

## Conclusions

Cardiac tamponade is a rare but fatal complication in which the patient presents with tachycardia, shortness of breath, and visible venous pulsations in the neck. It should be suspected in all patients exhibiting these symptoms after insertion or removal of IVC filters. It is important to manage these complications on an emergency basis with a multidisciplinary approach involving surgical, anesthesia, and cardiovascular teams.
